# Incidence and prevalence of coma in the UK and the USA

**DOI:** 10.1093/braincomms/fcac188

**Published:** 2022-09-01

**Authors:** Daniel Kondziella, Moshgan Amiri, Marwan H Othman, Ettore Beghi, Yelena G Bodien, Giuseppe Citerio, Joseph T Giacino, Stephan A Mayer, Thomas N Lawson, David K Menon, Verena Rass, Tarek Sharshar, Robert D Stevens, Lorenzo Tinti, Paul Vespa, Molly McNett, Chethan P Venkatasubba Rao, Raimund Helbok, Curing Coma Campaign Collaborators, Yama Akbari, Melanie Boly, Neha Dangayach, Brian Edlow, Brandon Foreman, Emily Gilmore, Flora M Hammond, J Claude Hemphill, Theresa Human, Lori Kennedy Madden, Shraddha Mainali, Geert Meyfroidt, Martin Monti, Risa Nakase-Richardson, Paul Nyquist, DaiWai Olson, Soojin Park, Jose Javier Provencio, Louis Puybasset, Aarti Sarwal, Lori Shutter, Briana Witherspoon, John Whyte, Wendy Ziai

**Affiliations:** Department of Neurology, Rigshospitalet, Copenhagen University Hospital, Blegdamsvej 9, DK-2100 Copenhagen, Denmark; Department of Clinical Medicine, University of Copenhagen, Copenhagen 2100, Denmark; Department of Neurology, Rigshospitalet, Copenhagen University Hospital, Blegdamsvej 9, DK-2100 Copenhagen, Denmark; Department of Neurology, Rigshospitalet, Copenhagen University Hospital, Blegdamsvej 9, DK-2100 Copenhagen, Denmark; Department of Neuroscience, Istituto di Ricerche Farmacologiche Mario Negri IRCCS, Milan 20156, Italy; Department of Physical Medicine and Rehabilitation, Spaulding Rehabilitation Hospital, Harvard Medical School, Boston, MA 02115, USA; Department of Neurology, Massachusetts General Hospital, Harvard Medical School, Boston, MA 02114, USA; NeuroIntensive Care, ASST di Monza, Monza 20900, Italy; School of Medicine and Surgery, Università Milano Bicocca, Milan 20100, Italy; Department of Physical Medicine and Rehabilitation, Spaulding Rehabilitation Hospital, Harvard Medical School, Boston, MA 02115, USA; Department of Neurology, New York Medical College, Valhalla, NY 10595, USA; College of Nursing, The Ohio State University, Columbus, OH 43210, USA; Division of Anaesthesia, University of Cambridge, Cambridge CB2 2QQ, UK; Department of Neurology, Neuro-Intensive Care Unit, Medical University of Innsbruck, Innsbruck 6020, Austria; Neuro-anesthesiology and Intensive Care Medicine, Sainte-Anne Hospital, Paris-Descartes University, Paris 75006, France; Experimental Neuropathology, Infection and Epidemiology Department, Institut Pasteur, Paris 75015, France; Department of Anesthesiology and Critical Care Medicine, The Johns Hopkins University School of Medicine, Baltimore, MD 21287, USA; Department of Neurology, The Johns Hopkins University School of Medicine, Baltimore, MD 21218, USA; Department of Neurosurgery, The Johns Hopkins University School of Medicine, Baltimore 21287, MD, USA; Department of Neuroscience, Istituto di Ricerche Farmacologiche Mario Negri IRCCS, Milan 20156, Italy; Department of Neurology, David Geffen School of Medicine at UCLA, Los Angeles, CA 90095, USA; Department of Neurosurgery, David Geffen School of Medicine at UCLA, Los Angeles, CA 90095, USA; College of Nursing, The Ohio State University, Columbus, OH 43210, USA; Division of Vascular Neurology and Neurocritical Care, Baylor College of Medicine and CHI Baylor St Luke's Medical Center, Houston, TX 77030, USA; Department of Neurology, Neuro-Intensive Care Unit, Medical University of Innsbruck, Innsbruck 6020, Austria

**Keywords:** brain injury, cardiac arrest, coma, consciousness, COVID-19

## Abstract

The epidemiology of coma is unknown because case ascertainment with traditional methods is difficult. Here, we used crowdsourcing methodology to estimate the incidence and prevalence of coma in the UK and the USA. We recruited UK and US laypeople (aged ≥18 years) who were nationally representative (i.e. matched for age, gender and ethnicity according to census data) of the UK and the USA, respectively, utilizing a crowdsourcing platform. We provided a description of coma and asked survey participants if they—‘right now’ or ‘within the last year’—had a family member in coma. These participants (UK *n* = 994, USA *n* = 977) provided data on 30 387 family members (UK *n* = 14 124, USA *n* = 16 263). We found more coma cases in the USA (*n* = 47) than in the UK (*n* = 20; *P* = 0.009). We identified one coma case in the UK (0.007%, 95% confidence interval 0.00–0.04%) on the day of the survey and 19 new coma cases (0.13%, 95% confidence interval 0.08–0.21%) within the preceding year, resulting in an annual incidence of 135/100 000 (95% confidence interval 81–210) and a point prevalence of 7 cases per 100 000 population (95% confidence interval 0.18–39.44) in the UK. We identified five cases in the USA (0.031%, 95% confidence interval 0.01–0.07%) on the day of the survey and 42 new cases (0.26%, 95% confidence interval 0.19–0.35%) within the preceding year, resulting in an annual incidence of 258/100 000 (95% confidence interval 186–349) and a point prevalence of 31 cases per 100 000 population (95% confidence interval 9.98–71.73) in the USA. The five most common causes were stroke, medically induced coma, COVID-19, traumatic brain injury and cardiac arrest. To summarize, for the first time, we report incidence and prevalence estimates for coma across diagnosis types and settings in the UK and the USA using crowdsourcing methods. Coma may be more prevalent in the USA than in the UK, which requires further investigation. These data are urgently needed to expand the public health perspective on coma and disorders of consciousness.

## Teaser

The epidemiology of coma is unknown because case ascertainment with traditional methods is difficult. Crowdsourcing observations from 30 387 family members, Kondziella *et al.* of the Curing Coma Campaign provide the first data on coma incidence and prevalence in the UK and the USA (pooled annual incidence, 201 coma cases/100 000 population).

## Graphical Abstract

**Graphical abstract:**
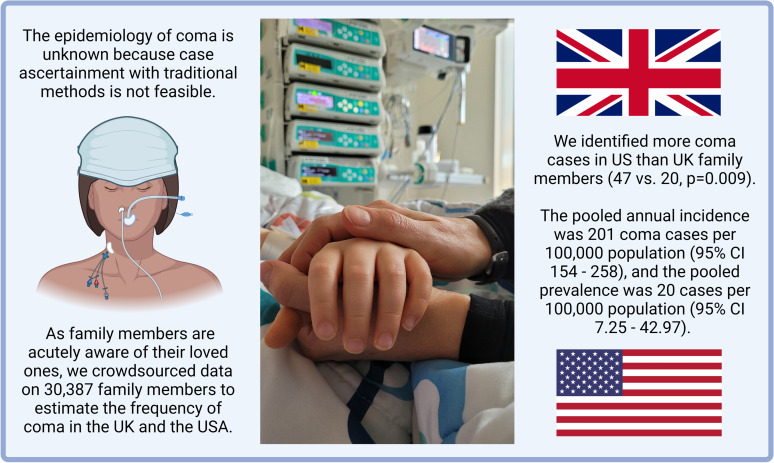

## Introduction

According to Plum and Posner, coma is a state of profound unawareness from which patients cannot be aroused; a normal sleep–wake cycle is absent; and the eyes are closed.^1^ Coma is a medical emergency commonly leading to death or unfavourable outcome if not promptly recognized and treated. It affects people worldwide regardless of age and social backgrounds, yet global epidemiological data for coma across diagnosis types and settings are unavailable.

In contrast to neurological conditions for which incidence and prevalence data are available,^2^ coma is not an aetiological diagnosis but a clinical syndrome that can result from a number of traumatic and non-traumatic brain injuries or systemic medical diseases.^1^ Coma is therefore not readily identifiable in registry studies based on electronic medical records or insurance billing codes. Given the typically short-lived nature of coma, resulting in either death or recovery of wakefulness within hours to weeks, there is also an unresolved debate within the medical community regarding how to define coma.^3^ In addition, the variability of clinical manifestations^4^ renders clinical surveillance studies with complete case ascertainment to establish nationwide epidemiological data infeasible. However, given the major implications of coma in terms of treatment and outcome, estimates of coma incidence and prevalence would be important to a wide range of medical and public decision-makers.^5^

In this study, we utilized a crowdsourcing approach to estimate the incidence and prevalence of coma in the UK and the USA. Our rationale was that, first, crowdsourcing methodology allows representative sampling of participants from the UK and the US populations based on official census data; and second, family members are acutely aware of the presence of coma impacting their loved ones. This suggests that given a precise definition, family members are able to state with high confidence when a relative has been in a coma within the preceding year (annual incidence) or is in a coma at the time of inquiry (point prevalence).

## Materials and methods

The objective of this study was to estimate the frequency of coma in the UK and USA. To this end, we collected data on the annual incidence (i.e. within the immediate 12 months prior to the survey) and the point prevalence (i.e. on the day the survey was conducted) of coma, reported by representative population samples from the UK and USA, matched for age, gender and ethnicity according to national census data from these two countries.

### Preregistration

We pre-registered the study protocol, including the objectives, on 11 October 2021 with the Open Science Framework (https://osf.io/jqan2), prior to data collection, to prevent p-hacking^6^ and HARKing (Hypothesizing After the Results are Known).^7^ We launched the survey on 19 October 2021 and closed it on 21 October 2021.

### Crowdsourcing

The survey vendor Prolific Academic (https://www.prolific.co) is an online crowdsourcing platform for the recruitment of human subjects that can be used for research purposes^8–13^ and that compares favourably to the Amazon’s Mechanical Turk in terms of data quality, including honesty and diversity of participants.^8^ In contrast to the Mechanical Turk, Prolific is for research purposes only and provides representative population samples from the UK and the USA (but no other countries). Briefly, the survey vendor takes the intended sample size and stratifies it across three demographics: age, sex and ethnicity, using census data from the US Census Bureau and the UK Office of National Statistics, respectively, to divide the sample into subgroups with the same proportions as the national population. This means, for example, that a representative sample contains the same proportion of 20- to 30-year-old Asian women as the national population (or as close as possible). The site has security checks to prevent infiltration by ‘bots’. Potential survey participants can be presented with a generic survey title (see below) to avoid influencing survey participation and study results.

This methodology has been used to investigate the prevalence of medical conditions in the general population,^9,10^ to identify biological markers of neurological phenomena^11,12^ and to assess for factors influencing the public opinion on ethical issues in medicine.^13,14^

### Study design

We performed an anonymous online cross-sectional survey with an unprimed sample of adult lay people (aged ≥18 years) from the UK and USA matched for age, gender and ethnicity according to UK and US census data, using the crowdsourcing platform Prolific Academic. Participants received a monetary reward upon completion of the survey in accordance with the platform’s ethical rewards principle ($9.60/h).

To reduce bias in an individual’s decision to respond to the survey, we chose a neutral title for the survey (‘The frequency of certain medical conditions’). We provided participants with an operational definition of coma and asked, if they—right now or within the last year—had a first- or second-degree family member in coma. We then inquired about the number of their first- and second-degree family members, which allowed us to calculate estimates of the incidence (‘within the past 12 months’) and the prevalence (‘right now’) of coma. In addition, we collected epidemiological data on the participants and their families, and for sensitivity analyses, we also inquired about coma cases in friends, neighbours, colleagues and the survey participants themselves. Table 1 displays an overview of the survey components. The study followed the Checklist for Reporting Of Survey Studies (CROSS) guideline; the complete survey including the CROSS is available from the Supplementary material.

**Table 1:** Questionnaire given to a representative sample of US and UK survey participants matched for age, gender and ethnicity according to national census data

How many first/second-degree family members do you have?^a^ How many of these first/second-degree family members live in the USA [UK]?^a^ At this very moment, do you have a first-degree family member who is in a coma?^b^ Who is the family member in a coma? How old is your family member in coma? What is the gender of your family member? Does your family member live in the USA [UK]? Is your family member admitted to a hospital in the USA [UK]? What is the condition that caused your family member to be in a coma?^c^ For how long has your family member been in a coma? Is your family member currently admitted to an intensive care unit? [If not:] (i) Does your family member require oxygen supplementation? (ii) Does your family member require nutritional support? (iii) Please describe your family member's physical condition with respect to breathing and feeding as well as you can. Do you have another first-degree family member who is in a coma at this moment? [Questions 3a-3i] Do you have a first-degree family member who has been in a coma within the past 12 months? [Questions 3a-3i] Did your family member survive? (i) Did your family member regain functional independence after coma? At this very moment, do you have a second-degree family member who is in a coma? [Questions 3a–3i] Do you have a second-degree family member who has been in a coma within the past 12 months? [Questions 3a–i] [Questions 5b] Do you personally know someone who is not first- or second-degree family (e.g. a friend, a work colleague, a neighbour) who is in a coma or who has been in a coma within the past year? [Questions 3a–3i]^d^ Have you ever been in a coma yourself? What was the condition that brought you into coma? Have you been in a coma more than once? Do you have diabetes (any type)? Do you have a cleft lip? Is a family member of yours taking this survey too?^e^

a Participants were first inquired about first-degree, then second-degree, family members. First-degree family members were defined as spouses, partners, children, parents, brothers and sisters or any humans that survey participants considered equivalent in terms of emotional and social importance; second-degree family member referred to grandparents, grandchildren, cousins, aunts, uncles or anyone else of similar emotional and social importance. Survey participants had to know with confidence if these people have been admitted to hospital within the preceding 12 months.

b See Materials and methods for coma definition.

c Options included traumatic brain injury, stroke, cardiac arrest, intoxication with illicit drugs, intoxication (any sort), brain infection (any sort), epilepsy, COVID-19 (primary reason for hospital admission), systemic infection other than COVID-19, liver failure, kidney failure, low or high blood sugar, low oxygen levels and/or low blood pressure, medically induced coma (i.e. a systemic illness that requires artificial coma to stabilize body functions), unknown and other.

d Here, ‘family member’ was replaced with ‘the patient’.

e This question was asked to avoid counting coma cases twice.

### Study population

Using the UK (ca. 67 million) and the US (ca. 328 million) population sizes, a confidence level of 95% and a margin of error of 5%, we estimated the required sample sizes to be 384 UK and 384 US participants (according to Krejcie and Morgan^15^). Since epidemiological data on coma are unknown, we aimed to enrol 2 × 1000 participants to identify an estimated number of 2–30 individuals with coma in each population sample. Inclusion criteria were adult UK/US citizens aged ≥18 years, registered with Prolific.co and willing to participate in this online survey; exclusion criteria were respondents with partial or incomplete surveys.

### Data collection

Demographic data collected included age, gender, ethnicity of participants and age and gender from their first- and second-degree relatives, as well as the exact number of first- and second-degree relatives for each participant (which was necessary to calculate the incidence and prevalence of coma cases identified relative to the population size sampled). Because this was a survey, we inquired about gender (which is a social construct, as opposed to biological sex).

For the purpose of this survey, ‘first-degree family members’ referred to a spouse or partner, children, parents, brothers and sisters and any other human that participants considered equivalent in terms of emotional and social importance to their life; ‘second-degree family’ included grandchildren, grandparents, cousins, aunts and uncles and anyone else of similar emotional and social importance. Participants were instructed that they were required to know with confidence whether or not these people had been admitted to the hospital within the preceding 12 months.

In the survey, we used the following coma definition for laypeople:

Coma is loss of consciousness due to impaired brain function. It typically has a sudden onset and always requires medical attention. There are many causes of coma including trauma, stroke, or cardiac arrest (when the heart stops and is then restarted). Coma is not caused primarily by medications, drugs, alcohol, or sedation. When people are in coma, they are not awake, cannot speak or move on their own, cannot answer questions or communicate in any way, are not aware of the world around them and cannot be awakened with stimulation. Occasionally, coma lasts only an hour or so until patients regain consciousness, but most of the time requires admission to the intensive care unit and the use of a breathing machine.

When participants indicated they knew someone who was in coma or had been in coma within the preceding 12 months, we collected various medical variables including the cause of coma, the duration of coma and the clinical outcome (see Supplementary material).

### Data quality assurance

Owing to caregiver distress, we thought that people with a family member in coma might be less likely to participate in online surveys than the average population, which would result in an underestimation of the true frequency of coma. For sensitivity analyses, we therefore also inquired about coma cases in more distant, i.e. second-degree, family members as well as in friends, neighbours, colleagues and the survey participants themselves. However, to avoid oversampling, we asked validation questions about the need for intensive care management, including artificial ventilation and nutritional support; we contacted survey participants directly via the survey vendor platform to confirm their data when in doubt; and we excluded coma cases that seemed implausible (e.g. patients hospitalized for ‘coma’ without the need for artificial ventilation or nutritional support). In addition, we used control questions to confirm the external validity of the survey data by asking if the survey participants had diabetes (which is common) or a cleft lip (which is rare) to compare the frequency of these two conditions with official UK and US census data.

### Data management and statistical analyses

The survey was designed using REDCap (https://projectredcap.org/resources/citations/), a secure HIPAA compliant electronic data capture system. Data were stored in REDCap in Denmark (the corresponding author’s country of residency). For descriptive statistics, baseline characteristics of participants and coma cases are presented as median values with interquartile ranges (IQRs) or mean values ± standard deviation (SD) for continuous variables and as frequencies and percentages for categorical variables. In univariate analysis, associations between potential predictors (age, gender, ethnicity) for coma were examined using the *χ*^2^ test and *t*-test for independent samples. The level of significance was set to 0.05 (two-sided) for all statistical tests. Statistical analysis was performed with R (R 3.6.1, R Development Core Team, Vienna, Austria). In a sensitivity analysis, data from the US sample were compared with that from the UK sample. The sample size calculation was performed as outlined above (‘Study population’).

### Ethics statement

The Institutional Review Board for Human Subject Research for Baylor College of Medicine and Affiliated Hospitals (BCMIRB), Houston, TX, USA, reviewed the research protocol and approved it by expedited procedures, which included a waiver of informed consent and a statement that this study does not require continuing review. Furthermore, in Denmark, the Copenhagen Ethics Committee waives approval for online surveys, literature reviews and online inquiries [section 14 (1) of the Committee Act. 2; http://www.nvk.dk/english]. The sponsor of the study was the Curing Coma Campaign (Neurocritical Care Society, Chicago, IL, USA^5^). As stated earlier, participants received a monetary reward upon completion of the survey.

### Data availability statement

Anonymized raw data can be accessed through the Supplementary material.

## Results

We distributed a survey (Table 1) to 2000 people, matched for age, gender and ethnicity according to UK/US census data and received 1971 (98.6%) valid responses: 994 from participants in the UK (mean age 45.2 ± 15.6 years; 51.7% women; 32.5% fully employed, 13.8% part-time employed, 6.8% unemployed, 13.6% retired/disabled, 5.0% other, 28.1% undisclosed employment status) and 977 from participants in the USA (mean age 44.8 ± 15.9 years; 52.1% women; 36.5% fully employed, 13.4% part-time employed, 9.8% unemployed, 14.9% retired/disabled, 4.3% other, 21.0% undisclosed employment status).

**Figure 1:**
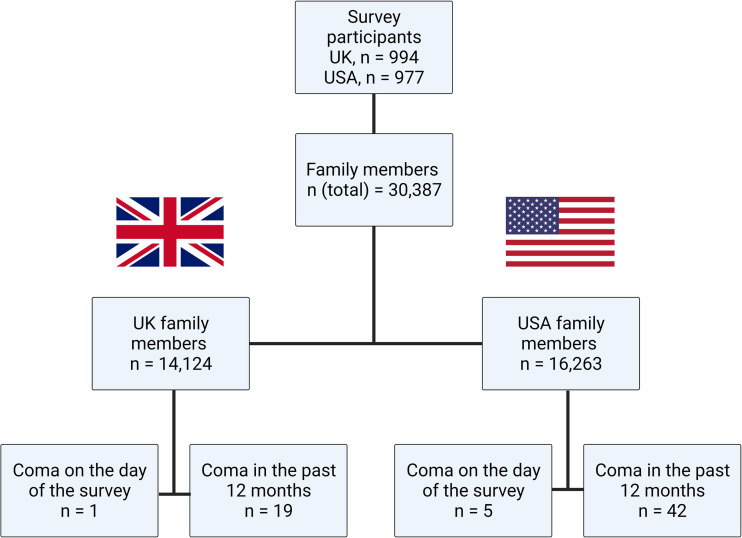
**Flowchart of the study survey**. Flowchart of the study survey, including coma cases identified in first- and second-degree family members on the day of the survey or within the preceding year.

The survey participants provided data on 30 387 first- and second-degree family members, of which 14 124 belonged to survey participants from the UK and 16 263 to participants from the USA. The UK participants had 5025 first-degree family members (4634 residing in the UK) and 9099 second-degree family members (7132 residing in the UK). The corresponding figures for participants from the USA were 5122 first-degree family members (4824 residing in the USA) and 11 141 second-degree family members (9650 residing in the USA). Figure 1 depicts a summary flow chart of the survey.

### Total coma cases identified

In total, 696 survey respondents (mean age 46.4 ± 14.8 years, 55.5% women) reported 737 coma cases. Of these, we excluded 23 patients who were residing outside the UK/US *and* were admitted to a hospital outside the UK/US (*n* = 21) or had implausible data (*n* = 2). The survey therefore identified a total of 714 plausible coma cases.

Individual patient-level data were available for 270 coma cases (Table 2), including 67 cases in first- and second-degree family members from the UK/USA (Supplementary Table 1), 38 coma cases in the survey participants themselves (see below) and 165 cases in ‘anyone now or within the past 12 months’. Coma was reported an additional 444 times in ‘anyone, anytime’.

**Table 2:** Characteristics of coma patients^a^

Group	Survey participants, self-reported (anytime)	Family members^b^	Non-family members^b^
Coma cases, *N*	38^c^	67	165
Country, *N* (%)			
USA	20 (52.6)	47 (70.1)	91 (55.2
UK	18 (47.3)	20 (29.9)	74 (44.8)
Age, mean (SD)	55.4 (18.3)	58.5 (21.9)	51.4 (18.2)
Gender, %^d^			
Female	51.4	43.3	33.3
Male	48.6	56.7	66.6
Cause of coma, *N* (%)			
Stroke	1 (2.6)	18 (26.9)	19 (11.5)
Medically induced	4 (10.5)	9 (13.4)	7 (4.2)
COVID-19	1 (2.6)	8 (11.9)	74 (44.8)
Cardiac arrest	9^e^ (23.7)	5 (7.5)	10 (6.1)
TBI	13^f^ (34.2)	5 (7.5)	22 (13.3)
Systemic infections	2 (5.4)	4 (6.0)	7 (4.2)
Intoxication	1 (2.6)	3 (4.5)	5 (3.0)
Unknown	0	1 (1.5)	6 (3.6)
Other^g^	7 (18.4)	14 (20.9)	15 (9.1)
Length of coma, days, median (range)	—	5 (1-150)	9 (1-372)
ICU admission, *N* (%)	—	59 (88.1)	159 (96.4)
Survival, *N* (%)			
Good outcome^h^	35 (100)	19 (28.3)	—
Bad outcome^h^	—	9 (13.4)	—
Death	—	33 (49.3)	—
Not determined	—	6 (8.9)	—

SD, standard deviation; TBI, traumatic brain injury; ICU, intensive care unit.

a For which patient data at the individual level were available.

b Comatose at the time the survey was done or within the preceding 12 months.

c Thirty-eight coma cases in 35 individuals as 2 had been comatose more than once.

d Because this was a survey, we inquired about gender (i.e. male, female, other) which is a social construct, as opposed to biological sex.

e Three episodes in one individual.

f Two episodes in one individual.

g Hypo-/hyperglycaemia, systemic infections, liver or kidney failure, brain infections and brain tumours.

h As defined by survey participants (‘functional independence’ versus non-independence).

### Annual incidence and point prevalence of coma in the UK

Of the 14 124 UK family members, we identified 1 coma case in a first-degree family member [0.007%, 95% confidence interval (CI) 0.00–0.04%; binomial proportion confidence interval) on the day the survey was conducted and 19 coma cases (7 first-degree and 12 second-degree family members; 0.13%, 95% CI 0.08–0.21%) within the preceding 12 months (Fig. 1). This translates to an annual incidence of 135 per 100 000 population (95% CI 81–210) and a point prevalence of 7 coma cases per 100 000 population (95% CI0.18–39.44) or 90 130 (95% CI 54 277–140 696) incidence cases and 4743 (95% CI 120–26 425) prevalence cases in the UK.

### Annual incidence and point prevalence of coma in the USA

Of the 16 263 US family members, we identified 5 coma cases (all first-degree family members; 0.031%, 95% CI 0.01–0.07%; binomial proportion confidence interval) on the day the survey was conducted and 42 coma cases (27 first-degree and 15 second-degree family members; 0.26%, 95% CI 0.19–0.35%) within the preceding 12 months (Fig. 1). This translates to an annual incidence of 258 per 100 000 population (95% CI 186–349) and a point prevalence of 31 coma cases per 100 000 population (95% CI 9.98–71.73) or 850 950 (95% CI 613 492–1 149 715) incidence cases and 101 303 (95% CI 32 895–236 360) prevalence cases in the USA.

### Frequencies of coma in the UK compared with the USA

The pooled annual incidence of coma for the UK/USA was 201 coma cases per 100 000 population (95% CI 154–258), and the pooled point prevalence was 20 coma cases per 100 000 population (95% CI 7.25–42.97).

Our survey identified more coma cases in US family members (*n* = 47) than in UK family members (*n* = 20; *P* = 0.009; *χ*^2^ test with Yates’ correction), although the number of self-reported coma cases was not statistically different between the two countries (see the next section).

Table 3 shows coma incidence and prevalence rates for the UK and USA compared with data from the Global Burden of Diseases, Injuries, and Risk Factors Study (GBD)^2^ for selected disorders frequently causing coma.

**Table 3:** Coma incidence and prevalence estimates for the UK and the USA compared with data from the GBD for disorders frequently causing coma

	UK		USA		Year, reference
	Incidence cases (95% CI)	Prevalence cases (95% CI)	Incidence cases (95% CI)	Prevalence cases (95% CI)	
Coma	90 130 (54 277–140 696)	4743 (120–26 425)	850 950 (613 492–1 149 715)	101 303 (32 895–236 360)	2021
Ischaemic stroke	49 376 (41 557–58 684)	575 501 (502 104–656 348)	310 274 (259 206–375 080)	5 871 392 (5 137 554–6 685 063)	2019^16^
Intracranial haemorrhage	13 603 (11 523–16 078)	85 877 (74 742–97 371)	71 731 (58 955–86 204)	663 770 (577 066–758 827)	2019^16^
Subarachnoid haemorrhage	11 208 (9520–13 306)	87 157 (74 443–103 448)	73 712 (60 842–88 992)	849 734 (706 672–1 013 293)	2019^16^
Meningitis	5664 (4588–6627)	n.d.	16 869 (14 990–18 661)	n.d.	2019^17^
Traumatic brain injury	168 579 (137 783–208 313)	382 133 (364 581–399 049)	1 110 578 (927 814–1 340 515)	2 349 017 (2 244 955–2 461 041)	2016^18^
Sepsis, all causes	245 783 (191 983–330 996)	n.d.	1 083 007 (884 243–1 342 025)	n.d.	2020^19^

Incidence cases denote novel diagnoses per year.

CI, confidence interval; GBD, Global Burden of Disease; n.d., no data available.

### Self-reported coma cases with full recovery

Thirty-eight coma events were self-reported (i.e. survey participants had been in a coma at least once in their life and recovered). These 38 self-reported coma episodes occurred in 35 participants, i.e. 2 participants had been in a coma twice and three times, respectively (Table 2). There was no statistical difference between the number of self-reported coma cases amongst survey participants from the UK compared with participants from the USA (18 versus 20 coma episodes; *P* = 0.83; *χ*^2^ test with Yates’ correction).

Given the survey participants’ mean age of 45 ± 15.75 years, this translates into 38 (95% CI 27–52; Poisson confidence interval incidence rate) self-reported coma cases with full recovery of consciousness in 90 000 life years (with ‘full recovery’ defined as being able to participate in the present survey) or ∼4 self-reported coma cases with full recovery per 10 000 life years.

### Aetiologies, duration of coma and survival rates

The five most common causes of coma in family members were stroke (*n* = 18, 26.9%), medically induced coma (*n* = 9, 13.4%), COVID-19 (*n* = 8, 11.9%), traumatic brain injury (*n* = 5, 7.5%) and cardiac arrest (*n* = 5, 7.5%). The remaining coma causes were systemic infections, hypo-/hyperglycaemia, intoxications, brain infections, liver or kidney failure and other or unknown causes (Table 2 and Supplementary Table 1). The median length of coma was 5 days (range 1–150), and 59 coma patients (88.1%) were admitted to the intensive care unit (ICU). Of the 61 coma patients with an established outcome (i.e. death or recovery of consciousness), 33 (54.1%) died, 9 (14.8%) survived with bad outcomes and 19 (31.1%) with good outcomes (as rated by survey participants according to ‘functional independence’ versus non-independence).

In non-family members in coma within the past 12 months, the five main coma causes were COVID-19 (*n* = 74; 44.8%), traumatic brain injury (*n* = 22; 13.3%), stroke (*n* = 19; 11.5%), cardiac arrest (*n* = 10; 6.1%) and medically induced coma (*n* = 7; 4.2%). The remaining coma causes were systemic infections, hypo-/hyperglycaemia, intoxications, brain infections, liver or kidney failure and other or unknown causes (Table 2). The median length of coma was 9 days (range 1–372 days), and 159 (96.4%) of the patients were admitted to the ICU.

Thirty-eight coma cases were reported in 35 survey participants (Table 2). The main causes of coma in this group were traumatic brain injury (*n* = 13; 34.2%), cardiac arrest (*n* = 9; 23.7%), medically induced coma (*n* = 4; 10.5%), systemic infections (*n* = 2; 5.4%) and hypo-/hyperglycaemia (*n* = 2; 5.4%).

Aetiologies, length of coma, outcomes and demographics of UK patients in coma were not different from US patients (all *P* > 0.09), see Supplementary Table 2 for details.

### Coma and COVID-19

For 83 individuals, COVID-19 was stated as the main cause of coma; 46 (55.4%) were US residents and 37 (44.5%) UK residents (mean age 53.7 ± 14.5 years; 28.9% women). The median duration of coma was 12 days (range 1–200 days), and 80 coma patients (96.4%) were admitted to the ICU. In non-family members, 74 of 165 (44.8%) were in coma due to COVID-19 within the past 12 months. The corresponding number for family members was 8 of 67 (11.9%) and for the responders themselves 1 of 38 (2.6%) coma cases.

### Survey quality control data

Sixty (6.0%) UK survey participants had diabetes, and 2 (0.2%) had a cleft lip; and 79 (8.1%) US participants had diabetes, and 1 had a cleft lip (0.1%). These figures are in line with prevalence data published elsewhere for diabetes (UK, 5.8%^20^; USA, 10.5%)^21^ and cleft lip (UK, 0.1%^22^; USA, 0.1%),^23^ indicating the responses were overall trustworthy.

Furthermore, the 65 survey participants who reported the 67 coma cases in first- and second-degree family members were comparable in terms of their demographics (53.8% females, mean age 42.4 ± 14.3 years) compared with all the 1971 survey participants (see above), indicating the absence of a systematic reporting bias associated with age or gender.

## Discussion

Prior to this work, epidemiological data on coma across diagnosis types and settings were unavailable. Authoritative texts on coma make no reference to the epidemiology of coma,^1,24^ and widely accessed medical online resources do not mention this aspect either^25^ or simply state that the epidemiology of coma depends on the aetiology.^26^ Here, we provide the first epidemiological estimates for coma in the UK and the USA.

The pooled incidence was 201 coma cases per 100 000 population per year, and the pooled prevalence was 20 coma cases per 100 000 population. Given that coma is a temporary condition typically lasting no more than two to three weeks,^1^ it makes sense that coma is relatively infrequent in terms of prevalence, while its incidence is comparable to conditions often encountered in intensive care such as sepsis or traumatic brain injury (Table 3).

Interestingly, we identified more coma cases in US than UK family members (47 versus 20). We do not think this difference reflects perceptional bias of the survey participants because the rate of self-reported coma was similar. It might be a statistical chance finding, but the *P*-value of 0.009 suggests otherwise. The cause of this difference would likely be multifactorial. Although speculative, one contributing factor might be a difference in pre-hospital and in-hospital triage systems between the two countries. For example, the number of ICU beds in the UK (6.6 ICU beds/100 000 people)^27^ is considerably lower than in the US (28 ICU beds/100 000 people),^28^ so it seems conceivable that the threshold to admit patients with a poor prognosis to the ICU (and maintain ICU support) is higher in the UK than in the USA, which might decrease the overall frequency of coma in the UK. Coincidence or not, the ratio of comatose UK and US family members on the day of the survey was very similar to the ratio of ICU beds in the two countries (1–5 versus 1–4.3). However, this suggestion requires further research to definitively determine a causal occurrence and possible contributing factors.

### Strengths and limitations

Crowdsourcing to obtain epidemiological data is an unconventional (albeit exponentially growing)^29^ approach that requires careful methodological consideration.

As stated earlier, coma cases are difficult to ascertain with traditional methods based on registry studies or prospective observational studies in hospital settings: coma occurs with myriad underlying conditions;^1^ it is a short-lasting, transitional state leading to the recovery of consciousness, death or a prolonged disorder of consciousness such as the unresponsive wakefulness syndrome^30^ or minimally conscious state,^31^ typically within hours to a few weeks; it may present with unusual features^4^; and its exact definition is disputed even amongst experts.^3^

For the reasons outlined and because family members are acutely aware of their loved ones’ wellbeing,^32,33^ we chose to rely on unprimed laypeople matched for age, gender and ethnicity according to UK and US census data to collect coma cases in family members. Of note, even though people who complete online surveys are not representative of the general population in unmeasurable ways (e.g. they are likely more technologically savvy than the average individual), in the present survey, this confound was mitigated by the fact that the primary objective of the survey was to collect data on family members, rather than individuals themselves. We therefore assumed that given precise instructions and a carefully drafted coma definition (see Materials and methods), family members would be able to identify relatives in coma reliably and with high accuracy.

Indeed, various sensitivity analyses suggest our data are overall robust. First, the prevalence estimates of diabetes and cleft lip in our cohort match official figures reported for the UK^20,22^ and the USA,^21,23^ and the UK figures for diabetes and cleft lip also confirmed UK figures obtained in an earlier unrelated crowdsourcing work,^9^ indicating the participants’ answers were trustworthy. Second, more second-degree family members than first-degree family members resided outside the UK/USA (which would be expected). Third, the vast majority of coma cases occurred within the ICU setting (again as expected). Fourth, COVID-19 patients in coma were older; the ratio of male to female (2.43 to 1) COVID-19 coma cases almost exactly matches the numbers in the literature,^34^ and it makes sense that the proportion of COVID-19 cases was greater in more peripheral social contacts such as friends, neighbours and colleagues (probably reflecting positive recall bias) compared with family members. Fifth, although comparable figures do not exist, the rate of self-reported coma cases with full recovery (4/10 000 life years) appears plausible. Sixth, the median length of coma (5 days), clinical outcomes including mortality rates, and the range and frequency of underlying conditions are consistent with clinical experience.^1^ Finally, the demographics of survey participants who reported a coma case were similar to the demographics of the entire pool of survey participants, indicating the absence of bias in survey responses related to age and gender.

Several limitations must be kept in mind. Crowdsourcing involves self-elected participants who sign up for surveys and receive a monetary reward. There was no monetary incentive in our study to report a coma case; however, since we instructed participants that their reimbursement was the same irrespective of whether they would report a coma case or not. Furthermore, although survey respondents were matched by age, race and gender to the general UK and US populations, this does not mean that their first- and second-degree relatives were evenly matched on these factors. Also, given that our definition of second-degree family members was broad, it could well be that survey respondents were more likely to remember to report distant family members that did have a coma, resulting in positive recall bias. In addition, the survey was limited to English-speakers and to people of a certain socioeconomic status with access to the technology required for online surveys. Finally, we were unable to examine and confirm each coma case by review of the medical charts of the respective patient. We did, however, attempt (and managed) to check each coma case by reaching out to every survey participant who reported a coma case, asking them to confirm the data they had provided, and we excluded two cases which we deemed implausible owing to inconsistent responses. Of note, 6 cases within the 67 coma cases of family members within the past year considerably exceeded the expected length of coma of typically no longer than 2–3 weeks. The exclusion of these cases would have resulted in lower estimates. However, although it may well be that some, if not all, of these six patients were no longer in a coma but had entered unresponsive wakefulness or minimally conscious states, it seems very likely that they were in a coma to begin with, so we decided to count these patients as coma cases. Also, although we defined coma as ‘not caused primarily by medications, drugs, alcohol or sedation’, from the list of possible coma causes our survey participants could choose ‘medically induced coma (i.e. your family member suffers from a systemic illness that requires artificial coma to stabilize his/her body functions)’. The latter is clearly different from e.g. the sedation-induced coma required for a minor surgery. However, we acknowledge this might be controversial. Given that medically induced coma belongs to a grey zone, we preferred to identity these cases (9 of 67 coma cases) rather than causing confusion in survey responders. Furthermore, one may also argue that coma could be defined as a condition that lasts at least >24 h or exclusively occurs in the ICU setting, but we opted for a somewhat broader definition (and included control questions about nutrition and oxygen). In sum, we decided to err on the side of specificity and excluded all reported coma cases which appeared doubtful.

### Conclusions and future directions

For the first time, the present work provides incidence and prevalence estimates for coma in the UK and the USA. While the prevalence of coma seems relatively low, the incidence of coma (2 in 1000 people per year) appears high when compared with commonly encountered conditions such as sepsis and traumatic brain injury. This is consistent with coma being an inherently critical condition and highlights the importance of recent initiatives to raise the awareness for coma.^5,35^ The data also suggest a striking difference between the frequency of coma in the UK and the USA, which might in part be explained by differences in the hospital triage systems in the two countries, but this needs replication and further explanation before definitive conclusions can be made. Replication of this survey at a later stage is also needed to adjust for the evolving COVID-19 pandemic. However, we suggest that a combination of traditional epidemiological methods, including prospective case ascertainment, with crowdsourcing of family observations might provide even more accurate epidemiological estimates of coma in the future. Further important steps include investigations of the variability in care and management of coma patients, including comparative effectiveness research, across countries. All these data are urgently needed to broaden the public health perspective on coma and disorders of consciousness.

## Supplementary Material

fcac188_Supplementary_Data

## Appendix

Contributing collaborators of the Curing Coma Campaign are as follows: Yama Akbari, Melanie Boly, Neha Dangayach, Brian Edlow, Brandon Foreman, Emily Gilmore, Flora M. Hammond, J. Claude Hemphill III, Theresa Human, Lori Kennedy Madden, Shraddha Mainali, Geert Meyfroidt, Martin Monti, Risa Nakase-Richardson, Paul Nyquist, DaiWai Olson, Soojin Park, Jose Javier Provencio, Louis Puybasset, Aarti Sarwal, Lori Shutter, Briana Witherspoon, John Whyte, Wendy Ziai. Other campaign participants are listed in Supplementary Table 3.

## Abbreviations

CI =: confidence interval
COVID-19 =: Corona Virus Disease 2019
CROSS =: Checklist for Reporting Of Survey Studies
GBDs =: Global Burden of Diseases
HARKing =: Hypothesizing After the Results are Known
ICU =: intensive care unit
IQR =: interquartile range
TBI =: traumatic brain injury
SD =: standard deviation
