# Supplementary material for: Incidence and prevalence of coma in the UK and the USA
Source: Brain Commun. 2022 Sep 1;4(5):fcac188. doi: 10.1093/braincomms/fcac188 (PMC9486895; doi:10.1093/braincomms/fcac188)
Supplement: fcac188_Supplementary_Data [file fcac188_Supplementary_Data.zip › Supplementary Tables.docx]

| **Supplementary Table 1. Coma cases in first- and second-degree family members, “now or within past 12 months”** | | | | | | | | | |
| --- | --- | --- | --- | --- | --- | --- | --- | --- | --- |
| **ID** | **Country of residency** | **Country of hospital admission** | **Age** | **Gender** | **Family relation** | **Cause of coma** | **Length of coma, days** | **ICU-admission** | **Survival *** |
| **74** | US | US | 32 | Female | Spouse | Low or high blood sugar | 125 | Yes | In coma on the day of the survey |
| **434** | US | US | 75 | Male | Parent | Brain infection | - | Yes | In coma on the day of the survey |
| **544** | US | US | 30 | Female | Spouse | Brain infection | 12 | Yes | In coma on the day of the survey |
| **660** | US | Non-US | 11 | Male | Child | Intoxication (other than alcohol and illicit drugs) | 23 | Yes | In coma on the day of the survey |
| **923** | US | US | 34 | Female | Spouse | Medically induced | 9 | Yes | In coma on the day of the survey |
| **923 (2)** | US | US | 23 | Female | Sibling | Medically induced | 7 | Yes | Yes, bad outcome |
| **14** | US | US | 42 | Male | Parent | Cardiac arrest | 2 | Yes | No |
| **17** | US | US | 60 | Male | Other | Stroke | 100 | Yes | Yes, good outcome |
| **20** | US | US | 68 | Female | Parent | COVID-19 | 8 | Yes | No |
| **102** | US | Non-US | 56 | Male | Parent | Intoxication (other than alcohol and illicit drugs) | 4 | No | No |
| **117** | US | US | 68 | Male | Parent | Liver failure | 5 | No | No |
| **120** | US | US | 26 | Female | Sibling | Low or high blood sugar | 3 | No | Yes, good outcome |
| **144** | US | US | 49 | Male | Parent | Systemic infection | 8 | Yes | No |
| **145** | US | US | 38 | Male | Sibling | COVID-19 | 70 | Yes | No |
| **215** | US | Non-US | 64 | Female | Parent | Liver failure | 1 | No | No |
| **268** | US | US | 69 | Female | Parent | Stroke | 9 | Yes | No |
| **312** | US | US | 53 | Male | Spouse | Medically induced | 20 | Yes | Yes, bad outcome |
| **328** | US | US | 27 | Male | Spouse | TBI | 60 | Yes | Yes, bad outcome |
| **382** | US | US | 59 | Female | Parent | Stroke | 33 | Yes | No |
| **447** | US | US | 58 | Female | Parent | Low or high blood sugar | 150 | Yes | Yes, good outcome |
| **489** | US | US | 67 | Male | Other | COVID-19 | 7 | Yes | No |
| **560** | US | US | 64 | Female | Parent | COVID-19 | 1 | Yes | Yes, good outcome |
| **652** | US | US | 49 | Male | Sibling | Brain tumor | 4 | Yes | No |
| **712** | US | US | 79 | Male | Spouse | Unknown | 1 | Yes | Yes, good outcome |
| **730** | US | US | 72 | Male | Other | Stroke | 2 | Yes | No |
| **853** | US | US | 55 | Female | Sibling | Intoxication (other than alcohol and illicit drugs) | 1 | Yes | Yes, good outcome |
| **879** | US | US | 95 | Female | Other | Medically induced | 8 | No | No |
| **884** | US | US | 55 | Male | Sibling | TBI | 1 | No | Yes, good outcome |
| **902** | US | US | 89 | Female | Parent | Systemic infection | 1 | No | No |
| **911** | US | US | 79 | Female | Sibling | Stroke | 6 | Yes | No |
| **913** | US | US | 58 | Female | Sibling | Stroke | 7 | Yes | No |
| **950** | US | US | 25 | Female | Child | Epilepsy | 1 | Yes | Yes, good outcome |
| **23** | Non-US | US | 55 | Female | Second degree | Stroke | 3 | No | No |
| **42** | US | US | 92 | Female | Second degree | Stroke | 2 | Yes | Yes, good outcome |
| **119** | US | US | 39 | Male | Second degree | Cardiac arrest | 10 | Yes | No |
| **232** | US | US | 75 | Male | Second degree | Cardiac arrest | 3 | Yes | Yes, good outcome |
| **277** | US | US | 82 | Female | Second degree | Stroke | 14 | Yes | Yes, bad outcome |
| **287** | US | US | 40 | Male | Second degree | Stroke | 10 | Yes | Yes, bad outcome |
| **312** | US | US | 60 | Male | Second degree | Hypoxia | 2 | Yes | Yes, good outcome |
| **371** | US | US | 56 | Male | Second degree | Stroke | 6 | Yes | Yes, good outcome |
| **373** | US | US | 81 | Male | Second degree | Stroke | 9 | Yes | Yes, good outcome |
| **388** | US | US | 79 | Male | Second degree | Systemic infection | 2 | Yes | No |
| **463** | US | US | 68 | Female | Second degree | COVID-19 | 7 | Yes | Yes, good outcome |
| **584** | US | US | 84 | Male | Second degree | Stroke | 5 | Yes | No |
| **833** | US | US | 74 | Male | Second degree | Stroke | 6 | Yes | No |
| **845** | US | US | 74 | Male | Second degree | COVID-19 | 3 | Yes | No |
| **851** | US | US | Newborn | Male | Second degree | Medically induced | 3 | Yes | Yes, good outcome |
| **306** | UK | UK | 26 | Female | Child | Stroke | 1 | Yes | In coma on the day of the survey |
| **35** | UK | UK | 57 | Male | Other | Medically induced | 1 | Yes | No |
|  | UK | UK | 67 | Female | Second degree | Stroke | 5 | Yes | No |
| **162** | UK | UK | 70 | Male | Parent | TBI | 14 | Yes | Yes, bad outcome |
| **168** | UK | UK | 51 | Female | Parent | Medically induced | 5 | Yes | Yes, bad outcome |
| **694** | UK | UK | 76 | Male | Parent | Other | 5 | Yes | Yes, good outcome |
| **776** | UK | UK | 78 | Female | Parent | Stroke | 4 | Yes | Yes, bad outcome |
| **791** | UK | UK | 78 | Male | Other | COVID-19 | 8 | Yes | No |
| **915** | UK | UK | 65 | Male | Sibling | Brain tumor | 1 | Yes | Yes, bad outcome |
| **32** | UK | UK | 91 | Female | Second degree | Liver failure | 1 | Yes | No |
| **33** | UK | UK | 4 | Male | Second degree | Systemic infection | 2 | Yes | Yes, good outcome |
| **115** | UK | UK | 56 | Male | Second degree | Medically induced | 2 | Yes | No |
| **150** | UK | UK | 89 | Male | Second degree | Cardiac arrest | 3 | Yes | No |
| **152** | UK | UK | 51 | Male | Second degree | Cardiac arrest | 3 | Yes | Yes, good outcome |
| **311** | UK | UK | 25 | Female | Second degree | TBI | 5 | Yes | Yes, good outcome |
| **478** | UK | UK | 83 | Male | Second degree | Stroke | 7 | Yes | No |
| **571** | UK | UK | 82 | Male | Second degree | COVID-19 | 7 | Yes | No |
| **620** | UK | UK | 64 | Male | Second degree | Brain infection | 2 | Yes | No |
| **726** | UK | UK | 65 | Female | Second degree | TBI | 2 | Yes | No |
| **924** | UK | UK | 55 | Female | Second degree | Medically induced | 1 | Yes | No |
| * Outcome of family members who survived was graded by the survey participants as good or bad according to “functional independence” vs. non-independence | | | | | | | | | |

| **Supplementary Table 2. Characteristics of all coma cases with individual patient data reported by UK vs. US survey participants** | | | |
| --- | --- | --- | --- |
|  | **US coma cases**  **N = 158** | **UK coma cases**  **N = 112** | **OR (p-value)**  **1.17 (0.22)** |
| Age, mean (SD) | 53.6 (19.1) | 53.6 (18.3) | p-value: 0.98 |
| Gender, N (%)   - Female - Male | 63 (39.9)  95 (60.1) | 41 (36.6)  71 (63.4) | 1.15 (0.61)  0.87 (0.61) |
| Coma cases, *N* (%) |  |  |  |
| - Family members, current | 5 (3.2) | 1 (0.9) | 4.34 (0.29) |
| - Family members, past 12 months | 42 (26.6) | 19 (17.0) | 1.9 (0.02)* |
| - Coma in non-family members | 91 (57.6) | 74 (66.0) | 1.06 (0.73) |
| - Self-reported coma cases | 20 (12.6) | 18 (16.1) | 1.12 (0.74) |
| Survival^¤^, N (%)   - All - Good outcome - Bad outcome | 20  15  5 | 8  4  4 | 1.24 (0.78)  2.87 (0.37)  0.35 (0.37) |
| Etiology of coma, N (%) |  |  |  |
| - COVID-19 | 46 (29.1) | 37 (33.0) | 0.83 (0.51) |
| - Stroke | 25 (15.8) | 13 (11.6) | 1.43 (0.42) |
| - TBI | 20 (12.7) | 20 (17.9) | 0.67 (0.31) |
| - Cardiac arrest | 15 (9.5) | 9 (8.0) | 1.20 (0.83) |
| - Medically induced | 12 (7.6) | 8 (7.1) | 1.07 (1.0) |
| - Intoxication other than alcohol and illicit drugs | 8 (5.1) | 1 (0.9) | 5.89 (0.09) |
| - Systemic infection | 5 (3.2) | 8 (7.1) | 0.43 (0.16) |
| - Hypo/hyperglycemia | 5 (3.2) | 1 (0.9) | 3.6 (0.41) |
| - Other or unknown | 22 (13.9) | 15 (13.4) | 1.05 (1.0) |
| Length of coma, days, Mean (SD) | 19.2 (35.5) | 22.9 (51.7) | p-value: 0.54 |
| ICU admission, N (%) | 129 (93.5) | 89 (94.7) | 0.81 (0.79) |
| *^¤^Family members in coma within the preceding past years – the survival rate of other coma groups is unknown.*  **Statistically significant (p<0.05)*  *Abbreviations:*  *SD; Standard deviation, TBI; Traumatic brain injury, ICU; Intensive care unit* | | | |

| **Supplementary Table 3.** **Acknowledgement of Curing Coma Campaign collaborators participating in the overall program** | | | |
| --- | --- | --- | --- |
| Venkatesh | Aiyagari | Salia | Farrokh |
| Yama | Akbari | Simona | Ferioli |
| Sheila | Alexander | Davinia | Fernandez-Espejo |
| Anne | Alexandrov | Joseph | Fins |
| Ayham | Alkhachroum | Oliver | Flower |
| Fawaz | Al-Mufti | Brandon | Foreman |
| Moshgan | Amiri | Romer | Geocadin |
| Brian | Appavu | Joe | Giacino |
| Meron | Awraris | Christie | Gibbons |
| Mary Kay | Bader | Emily | Gilmore |
| Ram | Balu | Ursula | Gorska |
| Megan | Barra | Olivia | Gosseries |
| Rachel | Beekman | Theresa | Green |
| Ettore | Beghi | Theresa | Green |
| Kathleen | Bell | David | Greer |
| Erta | Beqiri | Mary | Guanci |
| Tracey | Berlin | Cecil | Hahn |
| Thomas | Bleck | Ryan | Hakimi |
| Yelena | Bodien | Flora | Hammond |
| Varina | Boerwinkle | Daniel | Hanley |
| Melanie | Boly | Ahmed | Hassan |
| Alexandra | Bonnel | Raimund | Helbok |
| Emery | Brown | Claude | Hemphill |
| Eder | Caceres | Holly | Hinson |
| Josh | Cain | Karen | Hirsch |
| Elizabeth | Carroll | Sarah | Hocker |
| Emilio G. | Cediel | Peter | Hu |
| Sherry | Chou | Xiao | Hu |
| Giuseppe | Citerio | Andrew | Hudson |
| Jan | Claassen | Theresa | Human |
| Angela | Comanducci | David | Hwang |
| Chad | Condie | Matthew | Jaffa |
| Katie | Cosmas | Luke | James |
| Claire | Creutzfeldt | Anna | Janas |
| Neha | Dangayach | Morgan | Jones |
| Michael | DeGeorgia | Sheryl | Katta-Charles |
| Caroline | Der-Nigoghossian | Emanuela | Keller |
| Masoom | Desai | Maggie | Keogh |
| Michael | Diringer | Jenn | Kim |
| Brian | Edlow | Hannah | Kirsch |
| Satoshi | Egawa | Nerissa | Ko |
| Ari | Ercole | Daniel | Kondziella |
| Anna | Estraneo | Walter | Koroshetz |
| Guido | Falcone | Natalie | Kreitzer |
| Julie | Kromm | Llewellyn | Padayachy |
| Abhay | Kumar | Soojin | Park |
| Pedro | Kurtz | Melissa | Pergakis |
| Steven | Laureys | Leah | Phillips |
| Thomas | Lawson | Len | Polizzotto |
| Christos | Laziridis | Nader | Pouratian |
| Nicolas | Lejeune | Marilyn | Price Spivack |
| Ariane | Lewis | Lara | Prisco |
| John | Liang | Javier | Provencio |
| Geoff | Ling | Louis | Puybasset |
| Sarah | Livesay | Chethan | Rao |
| Andrea | Luppi | Verena | Rass |
| Lori | Madden | Risa | Richardson |
| Craig | Maddux | Cassia | Righy |
| Dea | Mahanes | Chiara | Robba |
| Shraddha | Mainali | Courtney | Robertson |
| Nelson | Maldonado | Benjamin | Rohaut |
| Rennan | Martins Ribeiro | John | Rolston |
| Marcello | Massimini | Mario | Rosanova |
| Stephan | Mayer | Eric | Rosenthal |
| Victoria | McCredie | Mary Beth | Russell |
| Molly | McNett | Gisele | Sampaio Silva |
| Jorge | Mejia-Mantilla | Leandro | Sanz |
| David | Menon | Simone | Sarasso |
| Geert | Meyfroidt | Aarti | Sarwal |
| Julio | Mijangos | Nicolas | Schiff |
| Dick | Moberg | David | Seder |
| Asma | Moheet | Vishank Arun | Shah |
| Martin | Monti | Angela | Shapshak |
| Chris | Morrison | Tarek | Sharshar |
| Amiri | Moshgan | Lori | Shutter |
| Susanne | Muehlschlegel | Jacobo | Sitt |
| Brooke | Murtaugh | Beth | Slomine |
| Lionel | Naccache | Peter | Smielewski |
| Masao | Nagayama | Wade | Smith |
| Girija | Natarajan | Sam | Snider |
| Virginia | Newcombe | Lennart | Spindler |
| Niklas | Nielsen | Emmanuel | Stamatakis |
| Naomi | Niznick | Alexis | Steinberg |
| Filipa | Noronha Falcão | Robert | Stevens |
| Paul | Nyquist | Jose | Suarez |
| DaiWai | Olson | Shaurya | Taran |
| Marwan | Othman |  |  |
| Adrian | Owen |  |  |
| May | Teresa |  |  |
| Aurore | Thibaut |  |  |
| Zachary | Threlkeld |  |  |
| Lorenzo | Tinti |  |  |
| Daniel | Toker |  |  |
| Michel | Torbey |  |  |
| Stephen | Trevick |  |  |
| Alexis | Turgeon |  |  |
| Andrew | Udy |  |  |
| Panos | Varelas |  |  |
| Paul | Vespa |  |  |
| Walter | Videtta |  |  |
| Henning | Voss |  |  |
| Ford | Vox |  |  |
| Amy | Wagner |  |  |
| John | Whyte |  |  |
| Briana | Witherspoon |  |  |
| Aleksandra (Sasha) | Yakhkind |  |  |
| Ross | Zafonte |  |  |
| Darin | Zahuranec |  |  |
| Chris | Zammit |  |  |
| Bei | Zhang |  |  |
| Wendy | Ziai |  |  |
| Lara | Zimmerman |  |  |
| Elizabeth | Zink |  |  |
